# 4,4′-[(1,3,4-Thia­diazole-2,5-di­yl)bis­(thio­methyl­ene)]dibenzonitrile

**DOI:** 10.1107/S1600536808022848

**Published:** 2008-07-26

**Authors:** Wenxiang Wang, Hong Zhao

**Affiliations:** a Ordered Matter Science Research Center, College of Chemistry and Chemical Engineering, Southeast University, Nanjing 210096, People’s Republic of China

## Abstract

The title mol­ecule, C_18_H_12_N_4_S_3_, consists of three essentially planar fragments, *viz*. two methyl-substituted benzonitrile rings and a substituted thia­diazole ring. The dihedral angles between the substituted benzonitrile rings and the central thia­diazole ring are 28.29 (10) and 78.83 (6)°, and the dihedral angle between the two benzonitrile rings is 72.89 (7)°.

## Related literature

For related literature, see: Tarafder *et al.*, (2000 ▶); El-Shekeil *et al.* (1988 ▶); Jinxia *et al.* (2003 ▶).
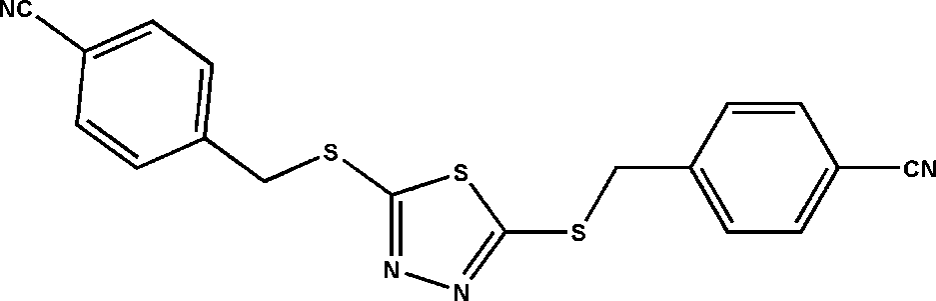


## Experimental

### 

#### Crystal data

C_18_H_12_N_4_S_3_
*M* *_r_* = 380.50
Monoclinic, 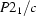

*a* = 7.6974 (15) Å
*b* = 8.4375 (17) Å
*c* = 27.272 (6) Å
β = 92.53 (3)°
*V* = 1769.5 (6) Å^3^
*Z* = 4
Mo *K*α radiation
μ = 0.43 mm^−1^
*T* = 293 (2) K
0.45 × 0.40 × 0.30 mm

#### Data collection

Rigaku Mercury2 diffractometer
Absorption correction: multi-scan (*CrystalClear*; Rigaku, 2005 ▶) *T*_min_ = 0.814, *T*_max_ = 0.903
15327 measured reflections
4028 independent reflections
2754 reflections with *I* > 2σ(*I*)
*R*_int_ = 0.043

#### Refinement

*R*[*F* ^2^ > 2σ(*F* ^2^)] = 0.053
*wR*(*F* ^2^) = 0.115
*S* = 1.05
4028 reflections
226 parameters
H-atom parameters constrained
Δρ_max_ = 0.20 e Å^−3^
Δρ_min_ = −0.25 e Å^−3^

### 

Data collection: *CrystalClear* (Rigaku, 2005 ▶); cell refinement: *CrystalClear*; data reduction: *CrystalClear*; program(s) used to solve structure: *SHELXS97* (Sheldrick, 2008 ▶); program(s) used to refine structure: *SHELXL97* (Sheldrick, 2008 ▶); molecular graphics: *SHELXTL* (Sheldrick, 2008 ▶); software used to prepare material for publication: *SHELXTL*.

## Supplementary Material

Crystal structure: contains datablocks I, global. DOI: 10.1107/S1600536808022848/fl2207sup1.cif

Structure factors: contains datablocks I. DOI: 10.1107/S1600536808022848/fl2207Isup2.hkl

Additional supplementary materials:  crystallographic information; 3D view; checkCIF report

## Figures and Tables

**Fig. 1.**
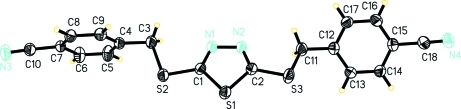
A view of the compound with the atomic numbering scheme. Displacement ellipsoids were drawn at the 30% probability level.

**Fig. 2.**
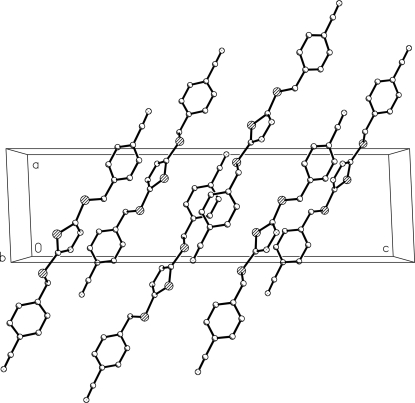
Packing diagram of the title compound, showing the structure along the b axis.

## supplementary crystallographic information

### Comment

1,3,4-thiadiazole-2,5-dithiol and its derivatives are interesting compounds that have attracted the attention of researchers because of their wide range of applications in many fields, such as the determination of trace elements, the synthesis of novel heterocyclic compounds with antimicrobial activity(El-Shekeil *et al.* 1988), advanced materials, and battery  cathodes (Jinxia *et al.*2003, Tarafder *et al.*, (2000)). In this paper, we report the structure of one such derviative, the title compound (I).

In (I,  Fig. 1) all bond lengths and angles are normal. There are three planar fragments in the molecule, *viz*. the central S1—S2—S3—C1—C2—N1—N2 plane with C1  farthest out at (0.0077 (19) Å)  and the two  benzonitrile systems C11—C18 and N4 with C11  farthest out at 0.0280 (19) Å) and C3—C19 and N3 with N3  farthest out at 0.0738 (21) Å. The dihedral angles  between the central substituted ring and benzonitrile systems are 28.29 (10)° and  78.83 (6)° and 72.89 (7)° between the two benzonitrile moieties. There is no evidence of typical hydrogenn bonding or  intermolecular π–π interactions.  Only  van-der-waals interactions are observed in the crystal is packing (Fig.2).

### Experimental

A dry 50 ml flask was charged with 1,3,4-thiadiazole-2,5-dithiol (10 mmol), 4-(bromomethyl)benzonitrile (20 mmol), K_2_CO_3_(10 mmol), and methanol (30 mL). The mixture was stirred with refluxing for 4 h and then was poured into water (40 ml), the precipitate was washed with water for 2–3 times and purified by recrystallization from methanol to give the crystals of the target material.

### Refinement

All the C—H hydrogen atoms were calculated geometrically and were allowed to ride on the C atoms to which they are bonded, with acyclic C—H distances ranging as 0.97 Å, phenyl C—H distances ranging as 0.93 Å, and with *U*_iso_(H) = 1.2Ueq(C).

### Crystal data

**Table:**

C_18_H_12_N_4_S_3_	*F*(000) = 784
*M**_r_* = 380.50	*D*_x_ = 1.428 Mg m^−^^3^
Monoclinic, *P*2_1_/*c*	Mo *K*α radiation, λ = 0.71073 Å
Hall symbol: -P 2ybc	Cell parameters from 12716 reflections
*a* = 7.6974 (15) Å	θ = 3.3–27.3°
*b* = 8.4375 (17) Å	µ = 0.43 mm^−^^1^
*c* = 27.272 (6) Å	*T* = 293 K
β = 92.53 (3)°	Prism, colorless
*V* = 1769.5 (6) Å^3^	0.45 × 0.40 × 0.30 mm
*Z* = 4	

### Data collection

**Table:**

Rigaku Mercury2 diffractometer	4028 independent reflections
Radiation source: fine-focus sealed tube	2754 reflections with *I* > 2σ(*I*)
graphite	*R*_int_ = 0.043
Detector resolution: 13.6612 pixels mm^-1^	θ_max_ = 27.5°, θ_min_ = 3.0°
CCD_Profile_fitting scans	*h* = −9→9
Absorption correction: multi-scan (*CrystalClear*; Rigaku, 2005)	*k* = −10→10
*T*_min_ = 0.814, *T*_max_ = 0.903	*l* = −35→35
15327 measured reflections	

### Refinement

**Table:**

Refinement on *F*^2^	Primary atom site location: structure-invariant direct methods
Least-squares matrix: full	Secondary atom site location: difference Fourier map
*R*[*F*^2^ > 2σ(*F*^2^)] = 0.053	Hydrogen site location: inferred from neighbouring sites
*wR*(*F*^2^) = 0.115	H-atom parameters constrained
*S* = 1.05	*w* = 1/[σ^2^(*F*_o_^2^) + (0.0435*P*)^2^ + 0.4638*P*] where *P* = (*F*_o_^2^ + 2*F*_c_^2^)/3
4028 reflections	(Δ/σ)_max_ < 0.001
226 parameters	Δρ_max_ = 0.20 e Å^−^^3^
0 restraints	Δρ_min_ = −0.25 e Å^−^^3^

### Special details

**Table:**

Geometry. All e.s.d.'s (except the e.s.d. in the dihedral angle between two l.s. planes) are estimated using the full covariance matrix. The cell e.s.d.'s are taken into account individually in the estimation of e.s.d.'s in distances, angles and torsion angles; correlations between e.s.d.'s in cell parameters are only used when they are defined by crystal symmetry. An approximate (isotropic) treatment of cell e.s.d.'s is used for estimating e.s.d.'s involving l.s. planes.
Refinement. Refinement of *F*^2^ against ALL reflections. The weighted *R*-factor *wR* and goodness of fit *S* are based on *F*^2^, conventional *R*-factors *R* are based on *F*, with *F* set to zero for negative *F*^2^. The threshold expression of *F*^2^ > σ(*F*^2^) is used only for calculating *R*-factors(gt) *etc*. and is not relevant to the choice of reflections for refinement. *R*-factors based on *F*^2^ are statistically about twice as large as those based on *F*, and *R*- factors based on ALL data will be even larger.

### Fractional atomic coordinates and isotropic or equivalent isotropic displacement parameters (Å^2^)

**Table:**

	*x*	*y*	*z*	*U*_iso_*/*U*_eq_	
C1	0.6555 (3)	0.3789 (3)	0.34124 (8)	0.0469 (5)	
C2	0.9214 (3)	0.3764 (3)	0.38800 (8)	0.0476 (6)	
C3	0.4395 (3)	0.2503 (3)	0.27094 (10)	0.0619 (7)	
H3A	0.5357	0.2535	0.2491	0.074*	
H3B	0.4455	0.1514	0.2891	0.074*	
C4	0.2700 (3)	0.2614 (3)	0.24188 (9)	0.0494 (6)	
C5	0.1254 (3)	0.1815 (3)	0.25715 (10)	0.0681 (8)	
H5	0.1344	0.1193	0.2853	0.082*	
C6	−0.0313 (3)	0.1926 (3)	0.23136 (10)	0.0675 (8)	
H6	−0.1277	0.1379	0.2419	0.081*	
C7	−0.0448 (3)	0.2853 (3)	0.18983 (8)	0.0463 (5)	
C8	0.0971 (3)	0.3662 (3)	0.17434 (8)	0.0504 (6)	
H8	0.0873	0.4293	0.1464	0.060*	
C9	0.2533 (3)	0.3540 (3)	0.20014 (9)	0.0521 (6)	
H9	0.3494	0.4087	0.1894	0.062*	
C10	−0.2123 (3)	0.3038 (3)	0.16434 (9)	0.0576 (7)	
C11	1.2030 (3)	0.2210 (3)	0.42467 (10)	0.0602 (7)	
H11A	1.1265	0.1432	0.4386	0.072*	
H11B	1.2195	0.1920	0.3908	0.072*	
C12	1.3756 (3)	0.2188 (3)	0.45268 (8)	0.0489 (6)	
C13	1.4027 (3)	0.2929 (3)	0.49739 (9)	0.0571 (6)	
H13	1.3126	0.3500	0.5106	0.068*	
C14	1.5604 (3)	0.2838 (3)	0.52286 (9)	0.0568 (6)	
H14	1.5767	0.3346	0.5530	0.068*	
C15	1.6945 (3)	0.1987 (3)	0.50351 (9)	0.0486 (6)	
C16	1.6682 (3)	0.1221 (3)	0.45909 (9)	0.0563 (6)	
H16	1.7575	0.0631	0.4462	0.068*	
C17	1.5102 (3)	0.1328 (3)	0.43390 (9)	0.0553 (6)	
H17	1.4936	0.0815	0.4039	0.066*	
C18	1.8610 (3)	0.1876 (3)	0.52986 (9)	0.0558 (6)	
N1	0.7442 (3)	0.2519 (3)	0.33425 (8)	0.0576 (5)	
N2	0.9002 (3)	0.2507 (2)	0.36166 (8)	0.0573 (5)	
N3	−0.3451 (3)	0.3216 (4)	0.14549 (9)	0.0848 (8)	
N4	1.9930 (3)	0.1766 (3)	0.54989 (9)	0.0740 (7)	
S1	0.75230 (9)	0.51067 (8)	0.38233 (2)	0.0594 (2)	
S2	0.45247 (8)	0.41683 (8)	0.31298 (2)	0.0595 (2)	
S3	1.10283 (9)	0.41341 (8)	0.42665 (3)	0.0642 (2)	

### Atomic displacement parameters (Å^2^)

**Table:**

	*U*^11^	*U*^22^	*U*^33^	*U*^12^	*U*^13^	*U*^23^
C1	0.0514 (13)	0.0435 (13)	0.0451 (13)	−0.0003 (11)	−0.0051 (10)	−0.0021 (10)
C2	0.0521 (14)	0.0403 (13)	0.0499 (13)	−0.0007 (11)	−0.0046 (10)	0.0007 (10)
C3	0.0557 (15)	0.0595 (16)	0.0689 (17)	0.0079 (13)	−0.0167 (12)	−0.0152 (13)
C4	0.0475 (13)	0.0459 (14)	0.0540 (14)	0.0037 (11)	−0.0076 (11)	−0.0056 (11)
C5	0.0654 (17)	0.0724 (19)	0.0653 (17)	−0.0093 (15)	−0.0122 (14)	0.0269 (14)
C6	0.0509 (15)	0.081 (2)	0.0694 (18)	−0.0132 (14)	−0.0042 (13)	0.0272 (15)
C7	0.0423 (13)	0.0497 (14)	0.0463 (13)	0.0028 (11)	−0.0039 (10)	−0.0007 (10)
C8	0.0535 (14)	0.0511 (14)	0.0464 (13)	−0.0022 (12)	−0.0002 (11)	0.0072 (11)
C9	0.0470 (14)	0.0515 (15)	0.0577 (15)	−0.0082 (12)	0.0031 (11)	−0.0010 (12)
C10	0.0511 (16)	0.0691 (18)	0.0523 (15)	0.0021 (14)	0.0005 (12)	0.0068 (13)
C11	0.0546 (15)	0.0506 (15)	0.0741 (17)	−0.0006 (12)	−0.0122 (13)	−0.0093 (13)
C12	0.0502 (14)	0.0396 (13)	0.0561 (15)	−0.0038 (11)	−0.0052 (11)	0.0033 (11)
C13	0.0517 (14)	0.0607 (16)	0.0584 (15)	0.0103 (13)	−0.0014 (12)	−0.0096 (13)
C14	0.0565 (15)	0.0585 (16)	0.0547 (15)	0.0084 (13)	−0.0059 (12)	−0.0061 (12)
C15	0.0464 (13)	0.0427 (13)	0.0564 (15)	−0.0002 (11)	−0.0037 (11)	0.0103 (11)
C16	0.0526 (15)	0.0525 (15)	0.0640 (16)	0.0085 (12)	0.0038 (12)	0.0001 (13)
C17	0.0603 (16)	0.0527 (15)	0.0526 (15)	0.0027 (13)	−0.0019 (12)	−0.0080 (12)
C18	0.0553 (16)	0.0512 (15)	0.0607 (16)	0.0061 (13)	0.0001 (12)	0.0081 (12)
N1	0.0526 (12)	0.0538 (13)	0.0648 (13)	0.0076 (10)	−0.0160 (10)	−0.0139 (10)
N2	0.0525 (12)	0.0532 (13)	0.0647 (13)	0.0061 (10)	−0.0147 (10)	−0.0131 (10)
N3	0.0503 (14)	0.126 (2)	0.0771 (17)	0.0071 (15)	−0.0094 (12)	0.0204 (16)
N4	0.0572 (14)	0.0870 (18)	0.0765 (16)	0.0146 (13)	−0.0119 (12)	0.0068 (13)
S1	0.0657 (4)	0.0441 (4)	0.0664 (4)	0.0074 (3)	−0.0187 (3)	−0.0101 (3)
S2	0.0572 (4)	0.0556 (4)	0.0640 (4)	0.0118 (3)	−0.0159 (3)	−0.0095 (3)
S3	0.0646 (4)	0.0468 (4)	0.0786 (5)	0.0012 (3)	−0.0278 (3)	−0.0094 (3)

### Geometric parameters (Å, °)

**Table:**

C1—N1	1.289 (3)	C9—H9	0.9300
C1—S1	1.725 (2)	C10—N3	1.134 (3)
C1—S2	1.742 (2)	C11—C12	1.503 (3)
C2—N2	1.287 (3)	C11—S3	1.799 (3)
C2—S1	1.727 (2)	C11—H11A	0.9700
C2—S3	1.740 (2)	C11—H11B	0.9700
C3—C4	1.499 (3)	C12—C13	1.378 (3)
C3—S2	1.814 (2)	C12—C17	1.382 (3)
C3—H3A	0.9700	C13—C14	1.374 (3)
C3—H3B	0.9700	C13—H13	0.9300
C4—C5	1.381 (3)	C14—C15	1.381 (3)
C4—C9	1.382 (3)	C14—H14	0.9300
C5—C6	1.372 (3)	C15—C16	1.380 (3)
C5—H5	0.9300	C15—C18	1.445 (3)
C6—C7	1.376 (3)	C16—C17	1.373 (3)
C6—H6	0.9300	C16—H16	0.9300
C7—C8	1.371 (3)	C17—H17	0.9300
C7—C10	1.446 (3)	C18—N4	1.136 (3)
C8—C9	1.370 (3)	N1—N2	1.386 (3)
C8—H8	0.9300		
			
N1—C1—S1	114.56 (17)	C12—C11—S3	111.49 (17)
N1—C1—S2	123.94 (18)	C12—C11—H11A	109.3
S1—C1—S2	121.49 (14)	S3—C11—H11A	109.3
N2—C2—S1	114.47 (17)	C12—C11—H11B	109.3
N2—C2—S3	124.36 (18)	S3—C11—H11B	109.3
S1—C2—S3	121.18 (14)	H11A—C11—H11B	108.0
C4—C3—S2	107.93 (16)	C13—C12—C17	118.7 (2)
C4—C3—H3A	110.1	C13—C12—C11	122.7 (2)
S2—C3—H3A	110.1	C17—C12—C11	118.6 (2)
C4—C3—H3B	110.1	C14—C13—C12	121.2 (2)
S2—C3—H3B	110.1	C14—C13—H13	119.4
H3A—C3—H3B	108.4	C12—C13—H13	119.4
C5—C4—C9	118.5 (2)	C13—C14—C15	119.6 (2)
C5—C4—C3	120.3 (2)	C13—C14—H14	120.2
C9—C4—C3	121.2 (2)	C15—C14—H14	120.2
C6—C5—C4	120.9 (2)	C16—C15—C14	119.7 (2)
C6—C5—H5	119.6	C16—C15—C18	119.9 (2)
C4—C5—H5	119.6	C14—C15—C18	120.4 (2)
C5—C6—C7	119.6 (2)	C17—C16—C15	120.0 (2)
C5—C6—H6	120.2	C17—C16—H16	120.0
C7—C6—H6	120.2	C15—C16—H16	120.0
C8—C7—C6	120.3 (2)	C16—C17—C12	120.7 (2)
C8—C7—C10	120.2 (2)	C16—C17—H17	119.6
C6—C7—C10	119.4 (2)	C12—C17—H17	119.6
C9—C8—C7	119.8 (2)	N4—C18—C15	178.6 (3)
C9—C8—H8	120.1	C1—N1—N2	112.22 (19)
C7—C8—H8	120.1	C2—N2—N1	112.35 (19)
C8—C9—C4	120.9 (2)	C1—S1—C2	86.40 (12)
C8—C9—H9	119.5	C1—S2—C3	99.23 (11)
C4—C9—H9	119.5	C2—S3—C11	98.82 (11)
N3—C10—C7	177.8 (3)		

## References

[bb1] El-Shekeil, A., Babaqi, A., Hassan, M. A. & Sheba, S. (1988). *Heterocycles*, **27**, 2577–2580.

[bb2] Jinxia, L., Zhan, H. & Zhou, Y. (2003). *Electrochem. Commun.* **5**, 555–560.

[bb3] Rigaku (2005). *CrystalClear* Rigaku Corporation, Tokyo, Japan.

[bb4] Sheldrick, G. M. (2008). *Acta Cryst.* A**64**, 112–122. 10.1107/S0108767307043930 18156677

[bb5] Tarafder, M. T. H., Azahari, K., Crouse, K. A., Yamin, B. M., Sundara Raj, S. S., Ibrahim, A. R. & Fun, H.-K. (2000). *Z. Kristallogr. New Cryst. Struct.* **215**, 487–488.

